# Is noxious stimulus-evoked electroencephalography response a reliable, valid, and interpretable outcome measure to assess analgesic efficacy in neonates? A systematic review and individual participant data (IPD) meta-analysis protocol

**DOI:** 10.1186/s13643-025-02890-4

**Published:** 2025-07-26

**Authors:** Luke Baxter, Marianne van der Vaart, Maria M. Cobo, Patricia Y. Gunawan, Karel Allegaert, Jonathan M. Davis, Mark Turner, Robert M. Ward, Edress Darsey, James P. Sheppard, Aomesh Bhatt, John van den Anker, An N. Massaro, Ramona L. Walls, Laura S. Song, Kanwaljit Singh, INC Pain Working Group, Dina Apele-Freimane, Takeshi Arimistu, Christine Barry, Deb Discenza, Olivia Giola, Collin Hovinga, Yamile Jackson, Danielle Matthews, Varsha Mehta, Katie Reginato Cascamo, Norma Vivas, Rebeccah Slater

**Affiliations:** 1 Department of Paediatrics, University of Oxford, Oxford, UK; 2 Colegio de Ciencias Biologicas y Ambientales, Universidad San Francisco de Quito USFQ, Quito, Ecuador; 3 Department of Development and Regeneration, KU Leuven, Leuven, Belgium; 4 Department of Pharmaceutical and Pharmacological Sciences, KU Leuven, Leuven, Belgium; 5 Department of Hospital Pharmacy, Erasmus Medical Center, Rotterdam, the Netherlands; 6 Department of Pediatrics, Tufts Medical Center, Boston, MA USA; 7 Department of Womens and Childrens Health, University of Liverpool, Liverpool, UK; 8 Department of Pediatrics, University of Utah School of Medicine, Salt Lake City, USA; 9 Pediatric Center of Excellence, Pfizer, NY USA; 10 Nuffield Department of Primary Care Health Sciences, University of Oxford, Oxford, UK; 11 Office of Pediatric Therapeutics, Food and Drug Administration (FDA), Silver Spring, MD USA; 12 Critical Path Institute, Data Collaboration Center, Tucson, AZ USA; 13 INC, Critical Path Institute (C-Path), Tucson, AZ USA; 14 Paediatric Neuroimaging Group Level2 Childrens Hospital, John Radcliffe Hospital, Oxford, UK; 15 Division of Clinical Pharmacology, Children’s National Hospital, Washington, DC USA; 16 Department of Child Health, Universitas Pelita Harapan, Tangerang, Indonesia

**Keywords:** Neonate, Pain, Brain, Electroencephalography, Biomarkers, Meta-analysis, Individual participant data, Validity, Reliability, Interpretability

## Abstract

**Background:**

There are several major challenges limiting our ability to test analgesic efficacy for treatment of neonatal pain, and progress in analgesic drug studies in neonates has stalled. One significant issue is the reliance of clinical pain assessments on traditional behavioural and vital signs-based measures and the exclusion of novel brain-based biomarkers. In this review protocol, we outline our strategy to assess the reliability, validity, and interpretability of an electroencephalography (EEG)-based response biomarker for assessment of acute somatic nociceptive pain in neonates.

**Methods:**

To standardise EEG analysis and generate the outcome of interest, we will perform an individual participant data (IPD) meta-analysis using data from neonates aged 34–44-week postmenstrual age that have had EEG recorded during acute somatic nociceptive skin-breaking procedures. Relevant data from both published and grey literature will be identified by searching six databases (MEDLINE, Embase, CINAHL, Web of Science, Scopus, Google Scholar), two clinical trial registry platforms (ClinicalTrials.gov, WHO ICTRP), and by consulting expert opinion. We will assess availability bias, data accuracy, and data quality by cross-referencing provided data with data descriptions in the literature, identifying duplicates and nonsensical values, and extracting quality control metrics. Data will be synthesised via a two-stage IPD meta-analysis using a random effects modelling approach grouped by site. Reliability (inter- and intra-rater) outcomes will be measured as Gwet’s AC1 coefficient. Validity (known-groups and known-stimuli) outcomes will be measured as EEG response magnitude differences between clinically meaningfully different stimuli. Interpretability will be addressed by providing normative values, in both original and standardised units.

**Discussion:**

The purpose of this study is to establish the reliability, validity, and interpretability of a specific EEG-based response biomarker for assessing acute somatic nociceptive pain in neonates. It will provide an overview of available data and how EEG is being used globally to assess acute neonatal pain. If sufficient IPD are made available and the outcome is reliable, valid, and interpretable, this work will support the use of EEG-based outcome measures as primary endpoints in clinical trials assessing analgesic efficacy in neonates.

**Systematic review registration**: The protocol was registered with PROSPERO on 14 July 2023: CRD42023444809.

**Supplementary Information:**

The online version contains supplementary material available at 10.1186/s13643-025-02890-4.

## Background

### Rationale

Hospitalised neonates undergo numerous painful procedures each day as part of routine clinical care, including heel lances, cannulation, intubation, and peripheral arterial/venous line insertions [1]. Unfortunately, analgesic provision for neonates is ad hoc, off-label, un-standardised, and suboptimal [2]. This is primarily due to difficulties in assessing acute pain in neonates due to the subjective nature of pain and the non-verbal nature of the neonatal population, with current pain outcome measures designed for older populations (children as well as adults) not being suitable. These challenges have limited our ability to test the efficacy and safety of analgesics for use in acute pain in this vulnerable population. As such, progress in analgesic drug studies in neonates has stalled [3].

There is a need for pain assessment tools in neonates that can accurately evaluate pain and that are specifically intended for use in this vulnerable patient population. More than 40 different multicomponent pain scales have been developed to assess neonatal pain [4]. This includes some that have been accepted by the US Food and Drug Administration (FDA) as trial endpoints (e.g. LNPS [Leuven Neonatal Pain Score], FLACC [Face, Legs, Activity, Cry, and Consolability], and Alder Hey Triage Pain Score) [5, 6]. However, there is a clear acknowledgement that these measures are only acceptable in the absence of better alternatives [7]. To advance the development and licensing of analgesics and improve the success of trials assessing analgesic efficacy, there is an urgent need for better (i.e. greater validity, reliability, and interpretability) primary endpoints to be developed for neonates.

In this review, we will assess the potential value of a brain-based pain-relevant response recorded using electroencephalography (EEG). A well-characterised EEG pattern of noxious stimulus-evoked brain activity exhibiting a positive deflection at approximately 560-ms post-stimulation has been recorded in peri-term-aged neonates in response to acute somatic nociceptive skin-breaking procedures such as heel lancing and intramuscular injections [8, 9]. The magnitude of this positive deflection can be quantified using a standardised waveform that is scaled to the amplitude of the evoked signal [10] using linear regression. This can be used to study the influence of analgesic interventions on the magnitude of noxious stimulus-evoked brain activity in randomised clinical trials [11, 12]. We suggest that this specific EEG metric of noxious stimulus-evoked brain activity can provide a reliable, valid, and interpretable proxy measure of neonatal pain that is not behaviour, autonomic, or haemodynamic based. This could serve as a useful endpoint to evaluate the efficacy of analgesics in neonatal clinical trials.

To assess the reliability, validity, and interpretability of noxious stimulus-evoked EEG activity in neonates for use as an analgesic efficacy endpoint in clinical trials, it is highly desirable to assess these properties across multiple geographic sites and independent research centres. Aggregating the data in the literature using meta-analysis is the gold standard for this research aim. While there are several research centres around the world that are working with neonatal noxious stimulus-evoked EEG data, there are a multitude of EEG pre-processing and analytic methods adopted in the literature. Only one research centre is currently using the EEG methodology outlined above [10]. Thus, a traditional aggregate data meta-analysis approach is not feasible. Instead, we will adopt the individual participant data (IPD) meta-analysis approach to request relevant raw EEG data that can subsequently be preprocessed and analysed using a standardised analysis pipeline. This will allow the derivation of a single standardised metric across all retrieved data.

To assess the reliability, validity, and interpretability of the EEG metric of neonatal acute pain assessment, we will adopt the COSMIN (COnsensus-based Standards for the selection of health Measurement INstruments) terminology framework [13, 14]. This terminology is closely related to that of the FDA [15]; the measurement properties of reliability and validity map directly, while the FDA measurement property ‘ability to detect change’ maps onto COSMIN’s measurement property ‘responsiveness’, which COSMIN classifies as a subcategory of validity [13]. However, the FDA framework has been developed with patient-reported outcome (PRO) measures in mind, while the specific EEG metric under investigation is a pharmacodynamic or response biomarker.

We have adopted the COSMIN framework due to its more expansive literature base, its broader coverage of both validity and reliability subtypes, and its greater clarity around the concept of measurement interpretability. We also provide a glossary in the supplementary information (Table S1) to clarify relevant terminology.

### Objectives

We aim to establish the extent to which the noxious stimulus-evoked EEG measure is a pain-relevant indicator that is suitable for use as a response biomarker in clinical trials of established and novel analgesics. To achieve this, we will assess its validity, reliability, and interpretability, as defined by the COSMIN taxonomy of measurement properties [13, 14] (see supplementary information Table S1: Glossary). Our primary research question is as follows: “Is the specific EEG-recorded brain activity magnitude metric under investigation, that is evoked by acute somatic nociceptive skin-breaking procedures in neonates 34–44 weeks PMA, a reliable, valid, and interpretable pain-relevant indicator?”. The research question is tabulated in PICO format in the supplementary information (Table S2).

When pre-processing EEG data, an initial quality assessment step involves determining whether to include or exclude an individual noxious stimulus-evoked EEG response (i.e. EEG epoch rejection) from the group-level analysis. This step involves a rater’s judgment, thus allowing us to assess rater reliability [16]. We will assess both inter-rater and intra-rater reliability based on independent raters’ decisions to include or exclude each stimulus–response epoch.

To assess validity [17], we focus on two types of construct validity: known-groups validity and known-stimuli validity (see glossary for definitions in Table S1). For known-groups validity, our ‘known groups’ should differ in the sensory intensity component of acute somatic nociceptive pain and are as follows: (i) noxious stimulation occurring with general anaesthetic or non-topical local anaesthetic (e.g. caudal block) and (ii) noxious stimulation without general anaesthetic or non-topical local anaesthetic. The mechanism of action of these anaesthetics is well-established and should reduce noxious stimulation input to the brain (topical local anaesthetics are not included since preliminary evidence suggests topical anaesthetics might not penetrate the skin deep enough to have an analgesic effect [18]). For known-stimuli validity, our ‘known stimuli’ that should differ in the sensory intensity component of acute somatic nociceptive pain are as follows: (i) acute somatic nociceptive skin-breaking procedures (e.g. heel lance) and (ii) acute somatic non-nociceptive non-skin-breaking procedures (e.g. sham heel lance). The differences between these two stimulus types should be reflected in the amount of noxious stimulation input reaching the brain.

Our research questions address the interpretability of values that can be considered at least moderately important in magnitude. As outlined in the validity assessments above, two key contexts that will be assessed are as follows: (i) a noxious skin-breaking procedure with and without anaesthetic (general anaesthetic or non-topical local anaesthetic) and (ii) a noxious skin-breaking procedure compared to a non-noxious non-skin-breaking procedure. Both scenarios compare relatively extreme cases where the magnitude of the effects will be much larger than a minimal important change (MIC). Assessing an MIC will not be possible, as there is no standard methodology to determine pain MICs for neonates. Whether the magnitude of the effects in the two scenarios is considered moderately or substantially important is open to interpretation.

To establish interpretability, an anchor-based approach [19] will be adopted relying on ‘clinician-based assessments’, which means that the interpretation of the two clinically important scenarios outlined above is deemed reasonable from a clinician’s (or researcher’s or parent’s) perspective, e.g. the difference between a noxious skin-breaking procedure and a non-noxious non-skin-breaking procedure is important from the perspective of clinicians, researchers, and parents. The anchor is the subjective assessment of the clinicians, researchers, and parents in our consortium, where the qualitative meaning of the quantitative values derives from the clinical meaning of the populations, stimuli, and interventions. To establish interpretability, typical values of the stimulus-evoked EEG response will be assessed to establish normative values for the neonatal population. The magnitude of the clinically important difference in stimulus-evoked EEG responses between noxious and innocuous stimuli will be determined, as will responses with or without anaesthetic. The effect sizes for these normative values and clinically meaningful differences will be reported to allow sample size planning for future randomised controlled trials. In addition to the anchor-based approach, the interpretability results will be supported by a statistics-based distribution-based approach [19]. We will also use a distribution-based approach to report small, medium, and large effect sizes using established statistical benchmarks.

In addition to the three primary objectives of assessing reliability, validity, and interpretability, there are three exploratory objectives to this study. The first is examining the potential differences in noxious stimulus-evoked EEG responses between biologically distinguishable groups, defined using variables such as age, sex, ethnicity, physiological state, and health status, the second is assessing the impact of proposed analgesic interventions (both pharmacological and non-pharmacological) and topical local anaesthetic interventions on the magnitude of the noxious stimulus-evoked EEG response, and the third objective is assessing the association between the noxious stimulus-evoked EEG response and the noxious stimulus-evoked response assessed using non-EEG methods (e.g. behavioural measures, physiological measures, infant clinical pain scales).

## Methods

### Protocol structure and registration

This protocol has been structured to conform to PRISMA-P guidelines [20, 21], and a completed PRISMA-P checklist is provided in the supplementary information. Our protocol was registered with PROSPERO on 14 July 2023: CRD42023444809. An expanded version of the protocol can also be found on the Open Science Framework (OSF) [22].

### Eligibility criteria

We include primary empirical studies with any study design that have EEG recordings during acute somatic nociceptive skin-breaking procedures for neonates aged 34–44 weeks PMA. There are no exclusion criteria based on language or year of dissemination. The eligibility criteria are tabulated in full in supplementary information Table S3.

### Information sources and search strategies

We use three categories of information sources: bibliographic databases, clinical trial registries, and authors of included studies. For electronic searches, the search strategy was initially developed in MEDLINE and then translated for other databases and registries. The search focused on the combination of three core topics: ‘neonate’, ‘pain’, and ‘EEG’. The search strategies were independently peer-reviewed using the PRESS checklist [23, 24] by an outreach librarian at the Bodleian Health Care Libraries, University of Oxford. The bibliographic databases (and their platforms) are MEDLINE (Ovid), Embase (Ovid), CINAHL (EBSCO Industries), Web of Science Core Collection (Clarivate Analytics), Scopus (Elsevier), and Google Scholar (Publish or Perish). Clinical trial registrations were identified using the following: ClinicalTrials.gov [25] and WHO ICTRP [26]. All search strategies are presented in full in the supplementary information.

### Study records

For the literature review data to identify sources of IPD, all screening (on title and abstract followed by full text) will be performed independently in duplicate, with disagreements resolved through discussion with a third reviewer. Data from bibliographic databases will be managed (deduplicated and screened) using EPPI-Reviewer Web [27], while data from clinical trial registries will be managed via spreadsheets and in-house scripts with data files stored safely on a university server (scripts will be publicly available via GitHub). Original investigators who have relevant EEG data will be invited to be co-authors on a scoping review, where the summary results of this literature search will be reported, and the accuracy of literature data extraction was verified by the original investigators.

After identifying original investigators with relevant data, we will also invite them to contribute their IPD for systematic review and meta-analysis and to be co-authors on the publication. All IPD from a single site will be pooled across studies conducted within the same site to form a single dataset per site (see supplementary information — Risk of Bias in Individual Studies). To ensure consistent analysis of all IPD across sites, a single researcher competent in implementing the specific analysis pipeline will visit all participating sites to run the analysis pipeline and extract the relevant outcomes and image files for each neonate’s dataset. Relevant non-EEG data (see the ‘Data items’ below) will be compiled locally by the participating sites, with central guidance. The IPD dataset will initially be stored locally at each participating site. The summary deidentified outcome measures per neonate and relevant non-EEG variables will be stored in spreadsheet files, shared with researchers at the University of Oxford, and stored securely on a University of Oxford server for subsequent meta-analysis. Image files for each EEG epoch will be stored with the spreadsheets. Finally, the spreadsheets and image files per site will be stored by C-Path on the Rare Disease Cures Accelerator-Data and Analytics Platform (RDCA-DAP), an FDA-funded initiative that provides a centralised and standardised data analytics infrastructure [28]. Each original investigator will also be invited to share their full IPD dataset with C-Path for storage on RDCA-DAP, to ensure safe and secure data storage that meets FAIR data principles [29, 30].

### Data items

For the literature review, the data items extracted from the literature will be outlined and defined in the data extraction form and accompanying data dictionary, which will be made publicly available as part of the scoping review publication.

For the IPD systematic review and meta-analysis, there are several variables required per individual EEG epoch for the meta-analysis. To address the validity and interpretability research objectives, a response magnitude (regression coefficient) and its associated measure of variance (the squared standard error of the regression coefficient) will be required. To address the reliability research objective, several epoch quality control (QC) metrics as well as image files of the EEG epochs will be required to guide users of the analysis methodology to make data-informed decisions regarding the inclusion or exclusion of each individual epoch. The specific details of how each of the response metrics and QC metrics are extracted will be provided in a forthcoming publication detailing the analysis pipeline. The analysis pipeline will be made publicly available via GitHub.

Additionally, non-EEG data will be required. To address the validity and interpretability research objectives, we will require information on the nature of the recorded noxious and innocuous stimuli (e.g. heel lance, sham heel lance) as well as whether the baby received general anaesthetic or non-topical local anaesthetic. For the exploratory analyses, we will require information relating to age, sex, ethnicity, physiological state, health status, analgesic interventions (both pharmacological and non-pharmacological), topical local anaesthetic interventions, and noxious stimulus-evoked responses assessed using non-EEG methods (e.g. behavioural measures, physiological measures, infant clinical pain scales). The exact data items required for the exploratory analyses will be based on the data extraction form from the scoping review and determined after data extraction for the scoping review is complete. This ensures that requests for data include items that are collected by the individual sites.

For full storage of the raw EEG data in C-Path’s RDCA-DAP, the exact data items required will be defined once data contributions are made and the full requirements are understood. Importantly, the storage of the raw EEG data in C-Path’s RDCA-DAP will not be a prerequisite for addressing all primary objectives of this IPD systematic review and meta-analysis.

### Outcomes and prioritisation

The three primary objectives (assessing reliability, validity, and interpretability) are of equally high priority. The three exploratory objectives (assessing biological groups, analgesic and topical anaesthetic interventions, and non-EEG measures) are of lower prioritisation.

Reliability outcomes will be measured as Gwet’s AC1 coefficient (a chance-corrected agreement coefficient and a more stable alternative to Cohen’s kappa), and 95% confidence intervals (CIs) will be reported. Adequacy will be assessed using the Altman’s benchmark scale: poor (− 1 to 0.2), fair (0.2 to 0.4), moderate (0.4 to 0.6), good (0.6 to 0.8), and very good (0.8 to 1).

Validity outcomes will be measured as stimulus-evoked EEG response magnitude differences (e.g. noxious minus innocuous, analgesic-negative minus analgesic-positive). Means and 95% CIs will be reported. Significance will be assessed by whether the 95% CI excludes (significant) or includes (not significant) the null value. Adequacy will be assessed using the COSMIN recommendations for hypothesis testing construct validity: at least 75% of the hypotheses should be confirmed.

Interpretability outcomes will be measured as stimulus-evoked EEG response magnitudes and magnitude differences. Means and 95% CIs will be reported. Statistical benchmarks of small, medium, and large effect sizes will supplement these outcomes, using established statistical benchmarks: Cohen’s D = 0.2, 0.5, and 0.8.

For the exploratory outcomes, biological group comparison outcomes will be measured as stimulus-evoked EEG response magnitudes (means and 95% CIs will be reported). Analgesic and topical local anaesthetic intervention assessment outcomes will be measured as stimulus-evoked EEG response magnitudes (means and 95% CIs will be reported). Associations between noxious stimulus-evoked EEG response and noxious stimulus-evoked response using non-EEG methods will be measured with Pearson correlation coefficients and 95% CIs. For composite or multicomponent infant pain scales (e.g. PIPP-R), we will assess associations between the EEG response and the overall infant pain scale score. If data availability allows, we will further assess associations between the EEG response and (i) each individual item in the scale and (ii) the mean value per dimension (e.g. behavioural dimension, physiological dimension) in the scale.

### Data synthesis

A two-stage IPD analytic approach will be adopted, because it automatically stratifies parameter estimates and residual variances appropriately by analysing each dataset separately in the first stage, utilises well-known meta-analysis methods that offer familiarity and transparency in the second stage, and enables visual summaries using forest plots [31]. Additionally, it provides the opportunity to assess the variability in primary outcomes by dataset. Where appropriate, a one-stage approach will be considered if most of the included datasets are small (e.g. < 20–30 participants) [31].

We will adopt a random effects modelling approach, as the assumption of a common effect across datasets is unlikely to be reasonable. For completeness, we will additionally report the fixed-effect (i.e. common-effect) result, with the random effects being the primary outcome.

When fitting the random effects model in the second stage, we will use the REML (restricted maximum likelihood) estimation approach and CIs generated using the HKSJ (Hartung-Knapp-Sidik-Jonkman) method [32]. This will be implemented in R [33] using the metafor package [34].

Summary effects will be generated for measures of reliability (inter- and intra-rater agreement), validity (magnitude differences for both known-groups and known-stimuli scenarios), and interpretability (typical magnitude and magnitude difference values), thus summarising the measurement properties for the specific EEG metric under investigation. From these summary effects, standardised effect sizes (Cohen’s D) will be generated, which can be used in future clinical trial sample size planning. Heterogeneity across datasets will be reported using *I*^2^, *τ*^2^, and 95% prediction intervals.

### Risk of bias in individual studies, meta-bias, and confidence in cumulative evidence

Given the nature of the research objectives (establishing novel outcomes of measurement properties and interpretability) and our level of data grouping (data grouped per site rather than per publication), the standard approaches to assessing bias and meta-bias are not directly applicable to this review. We provide an extended discussion of these topics in the supplementary information, where the aim to assess availability bias, data accuracy, and data quality is outlined. Similarly, given the nature of the research objectives, we will not formally assess the confidence in cumulative evidence.

## Discussion

The purpose of this systematic review and IPD meta-analysis is to establish the reliability, validity, and interpretability of a specific EEG-based response biomarker [10] for assessing acute somatic nociceptive pain in neonates. It will provide an overview of the available data and how EEG is being used globally to assess acute neonatal pain. If sufficient, IPD are made available, and the outcome is demonstrated to be reliable, valid, and interpretable; this work will support the use of EEG-based outcome measures as primary endpoints in clinical trials assessing analgesic efficacy in neonates. Currently, brain-based metrics are not featured in any of the dozens of clinical pain scales for neonatal pain [35], and we believe this review will help address this key knowledge gap.

This review is limited in its ability to establish certain types of reliability, validity, and interpretability, due to the nature of IPD meta-analysis, the nature of pain, and the non-verbal nature of neonates. The following discussion on reliability and validity subtypes and the interpretability of MICs is based on the COSMIN terminology [16, 17, 19].

Given that the EEG metric is a single-item outcome measurement, the internal consistency subtype of reliability is not relevant. Additionally, the reliability subtypes of measurement error and test–retest reliability are not possible. These reliability subtypes rely on the assumption that the patient conditions remain unchanged across repeated applications of painful stimulation (test–retest reliability) — an assumption that is highly questionable and cannot be established — or require that total measurement variance can be partitioned into true biological variance and noise variance (measurement error) — a requirement that cannot be met due to the inherent inability to disambiguate these variance subtypes.

Due to the quantitative and data-driven nature of an IPD meta-analysis, assessment of content validity is outside the review’s remit. Content validity (including face validity) is primarily qualitative and theory-driven and can be established using approaches such as questionnaires and expert panel reviews. Due to the subjective nature of pain and the non-verbal nature of neonates, assessing criterion validity is not possible as this type of validity requires the existence of a ‘gold standard’. Neither brain-based (e.g. EEG), behaviour-based, nor vital signs-based measures constitute a gold standard for pain assessment. As this review aims to establish measurement properties and interpretability of a specific single-item EEG metric, assessing structural validity is not relevant. Cross-cultural validity is not relevant, as there is no aspect of the specific EEG metric analysis approach that is language-based that would require translation and cross-cultural validation. Importantly, cross-cultural validity is unrelated to cultural differences in pain experience and response. Thus, there may be cross-cultural differences in noxious stimulus-evoked EEG signal magnitudes, and these potential differences can be explored in this review. However, exploring these potential cultural differences would not constitute an assessment of cross-cultural validity.

We also do not assess convergent validity, as establishing the agreement of the specific EEG metric with validated neonatal clinical pain scales (e.g. PIPP-R) should not be a prerequisite for validating an EEG metric. Given that no existing neonatal pain scale has reached gold standard status and given the mechanistic differences underpinning behavioural, vital signs, and EEG signals, these differing measures may indicate different pain-relevant signals that need not agree. Instead of assessing convergent validity, we will explore (as outlined in our “Methods” section) the association between the EEG metric and alternative non-EEG measures to help begin to shed light on the degree of overlap among these different measures. Divergent validity is unlikely to be able to be evaluated, as it would require researchers to have used a validated scale during the painful procedure to measure a concept of interest that is not pain.

As described in the interpretability objectives outlined in the “Background” section, there is no existing agreed-upon method to determine pain MICs for neonates. Consequently, it cannot be established by this review. However, the scenarios that we will assess compare relatively extreme cases, where the magnitude of the effects will be much larger than an MIC, thus allowing us to establish an upper limit to the magnitude of an MIC.

Lastly, this review is limited to assessing the reliability, validity, and interpretability of a noxious stimulus-evoked EEG response metric in neonates aged 34–44 weeks PMA. Infants below 34 weeks PMA display noxious stimulus-evoked brain activity of a different morphology than the brain activity quantified by the standardized waveform studied here [36, 37]. The standardised waveform used in this review has been developed for and used in neonates in this 34–44 week PMA age group [10, 12, 38–40]. While this age group does not include the youngest premature neonates to which most painful procedures happen, the methodology assessed in this review will help establish a foundation and road map for the future development of novel age-appropriate EEG outcome measures. This review will thus be of importance and interest to those hoping to improve pain assessment and treatment in the youngest most vulnerable neonatal age groups.

The dissemination plan is to publish results in a peer-reviewed academic journal. Any amendments made to this protocol when conducting the study will be outlined and reported in the final manuscript as well as an updated version of our extended protocol document on OSF.

## Supplementary Information

Supplementary Material 1.

## Data Availability

Not applicable.

## Abbreviations

CI: Confidence interval
COSMIN: COnsensus-based Standards for the selection of health Measurement INstruments
C-Path: Critical Path Institute
EEG: Electroencephalography
FAIR: Findability, accessibility, interoperability, and reusability
FDA: US Food and Drug Administration
FLACC: Face, Legs, Activity, Cry, and Consolability scale
HKSJ: Hartung-Knapp-Sidik-Jonkman
IPD: Individual participant data
LNPS: Leuven Neonatal Pain Score
MIC: Minimal important change
OSF: Open Science Framework
PICO: Population, intervention, comparator, and outcome
PIPP-R: Premature Infant Pain Profile-Revised
PMA: Postmenstrual age
PRESS: Peer Review of Electronic Search Strategies
PRISMA-P: Preferred Reporting Items for Systematic reviews and Meta-Analyses Protocols
PRO: Patient-reported outcome
PROSPERO: International Prospective Register of Systematic Reviews
QC: Quality control
RDCA-DAP: Rare Disease Cures Accelerator-Data and Analytics Platform
REML: Restricted maximum likelihood
WHO ICTRP: World Health Organization International Clinical Trials Registry Platform
