# Supplementary material for: Is noxious stimulus-evoked electroencephalography response a reliable, valid, and interpretable outcome measure to assess analgesic efficacy in neonates? A systematic review and individual participant data (IPD) meta-analysis protocol
Source: Syst Rev. 2025 Jul 26;14:152. doi: 10.1186/s13643-025-02890-4 (PMC12296588; doi:10.1186/s13643-025-02890-4)
Supplement: Supplementary file 1 — Supplementary Material 1. [file 13643_2025_2890_MOESM1_ESM.pdf]

Supplementary Information

Is noxious stimulus-evoked electroencephalography response a reliable, valid, and interpretable outcome measure to assess analgesic efficacy in neonates? A systematic review and individual participant data (IPD) meta-analysis protocol

Baxter et al.

Supplementary Tables

Table S1: Glossary

**Age terminology**	
Gestational age (GA)	Time from first day of last normal menstrual period to date of birth, usually expressed in completed weeks; GA is determined at birth using methods such as “best obstetrical estimate” (including first day of last menstrual period, physical examination of mother, prenatal ultrasound, history of assisted reproduction) and specific postnatal physical examinations. When a pregnancy has been achieved by assisted reproductive technology, GA is calculated from two weeks before the date of conception. [1]
Postnatal age (PNA)	Age calculated from date of birth (days, weeks, months, or years). (Also known as chronological age.) [1]
Postmenstrual age (PMA)	Time from first day of last normal menstrual period to day of assessment, that is, gestational age plus postnatal age, usually expressed in weeks. [1]
**Neonate terminology**	
Neonate	Neonates include term, post-term and preterm newborn infants. [1, 2]
Term neonate	37-42 weeks of GA [1, 2]
Preterm neonate	< 37 weeks of GA (equal or less than 36 weeks and 6 days of gestation) [1, 2]
Post-term neonate	> 42 weeks of GA [1, 2]
Neonatal period	The neonatal period for term and post-term newborn infants is defined as the day of birth plus 27 days. The neonatal period for preterm newborn infants is defined as the day of birth through the expected date of delivery plus 27 days. [1, 2]
**Pain terminology**	
Pain	An unpleasant sensory and emotional experience associated with, or resembling that associated with, actual or potential tissue damage [3]<br><br>*Note: The heel lance procedure and circumcision are the acute pain models for clinical trials in the neonate population recommended by the Analgesic, Anesthetic, and Addiction Clinical Trial Translations, Innovations, Opportunities, and Networks (ACTTION) Pediatric Pain Research Consortium consensus meeting* [4]
Nociceptive pain	Pain that arises from actual or threatened damage to non-neural tissue and is due to the activation of nociceptors [3]

Nociceptor	A high-threshold sensory receptor of the peripheral somatosensory nervous system that is capable of transducing and encoding noxious stimuli [3]
Nociception	The neural process of encoding noxious stimuli [3]
Noxious stimulus	A stimulus that is damaging or threatens damage to normal tissues [3]<br><br>*Note: In this study, we use an acute somatic nociceptive skin-breaking (and thus noxious) procedure as the noxious stimulus. The heel lance procedure (along with circumcision), which is a skin-breaking noxious stimulus, is the acute pain model for clinical trials in the neonate population recommended by the Analgesic, Anesthetic, and Addiction Clinical Trial Translations, Innovations, Opportunities, and Networks (ACTTION) Pediatric Pain Research Consortium consensus meeting* [4]
Innocuous stimulus	A stimulus that is not damaging and does not threaten damage to normal tissues.<br><br>*Note: In this study, we use acute somatic stimuli that are neither nociceptive nor skin-breaking (and thus non-noxious) as the innocuous stimulus.*
**Measurement terminology**	
Concept of Interest	The concept that the outcome assessment is intended to measure [5]
Outcome Assessment	A measuring instrument that provides a rating or score (categorical or continuous) that is intended to represent some aspect of the patient's medical status. Appropriate outcome assessments may include both clinical outcome assessment and biomarkers. [5]
Biomarker	A patient assessment that is not influenced by the patient's motivation or volition or a rater's judgment. [5]
Pharmacodynamic/Response biomarker	A biomarker used to show that a biological response has occurred in an individual who has been exposed to a medical product or an environmental agent. [6]
**Measurement property terminology**	
Validity	The degree to which an instrument measures the construct(s) it purports to measure [7, 8]
Construct validity	The degree to which the scores of an instrument are consistent with hypotheses based on the assumption that the instrument validly measures the construct to be measured [7, 8]
Known-groups validity	The degree to which an instrument discriminates between two groups known to differ on the construct of interest [7–9]

	Note: Synonyms include discriminative validity and extreme-groups validity.
Known-stimuli validity	The degree to which an instrument discriminates between two stimuli known to differ on the construct of interest.<br><br>*Note: We have defined known-stimuli validity as a variation on known-groups validity. Thus, a potential synonym could include extreme-stimuli validity.*
Reliability (domain)	The degree to which the measurement is free from measurement error [7, 8]
Reliability (measurement property)	The proportion of the total variance in the measurements which is because of true differences among patients [7, 8]
Inter-rater reliability	The extent to which two or more raters, assessing independently, are consistent with each other. [7, 8]
Intra-rater reliability	The extent to which a single rater is consistent with themselves on two or more separate occasions. [7, 8]
Interpretability	The degree to which one can assign qualitative meaning (that is, clinical or commonly understood connotations) to an instrument's quantitative scores or change in scores. [7, 8]
Anchor-based approach (to interpretability)	Uses an external criterion, or anchor, to determine what is considered as important improvement or important deterioration. [7, 8]
Distribution-based approach (to interpretability)	Based on distributional characteristics of the sample, and express the observed change in the measurement instrument under study to some form of variation to obtain a standardized metric [7, 8]

Table S2: Primary research question in PICO format

**Population**	Human neonates 34-44 weeks PMA
**Intervention**	Acute somatic nociceptive skin-breaking procedure
**Comparator**	The comparator intervention is dependent on the specific objective. For reliability assessments, there is no comparator intervention. For both validity and interpretability assessments, one assessment involves an acute non-noxious non-skin-breaking comparator intervention.
**Outcome**	EEG measures of intervention-evoked brain activity

Table S3: Eligibility criteria

**Inclusion criteria**	
**Population**	Human neonates 34-44 weeks PMA
**Intervention**	Acute somatic nociceptive skin-breaking procedure
**Comparator**	n/a
**Outcome**	EEG measures of intervention-evoked brain activity
**Study design**	Primary empirical studies with any study design e.g. interventional, observational, controlled, uncontrolled.
**Report characteristics**	Primary empirical research. All report types e.g. peer-reviewed publications, grey literature. Any year of dissemination. Any publication language.
**Exclusion criteria**	
**Population**	Humans younger than 34 weeks PMA and older than 44 weeks PMA; non-humans
**Intervention**	Non-acute noxious conditions, non-somatic noxious conditions, non-skin-breaking procedures
**Comparator**	n/a
**Outcome**	Studies that do not include EEG measures of brain activity evoked by acute somatic nociceptive skin-breaking procedures
**Study design**	None
**Report characteristics**	Secondary literature e.g. reviews, book chapters. Non-empirical research e.g. commentaries, opinions, perspectives.

Search strategies

For searching our population-of-interest, we used the "foetus and baby" search filter, which is an "Age Specific Filter" from the ISSG Search Filters Resource: https://sites.google.com/a/york.ac.uk/issg-search-filters-resource/home/age-groups

This (Ovid Medline) filter is published on the Canadian Health Libraries Association website: https://extranet.santecom.qc.ca/wiki/!biblio3s/doku.php?id=concepts:foetus-et-bebe

We modified this search filter to exclude irrelevant terms related to "foetus" and "pregnancy". The full original search filter is copied below, with terms that are irrelevant for our search displayed with strikethrough, and were thus deleted from our search:

> perinatal* OR ~~antepartum OR ante-partum OR intrapartum OR intra-partum~~ OR neonatal* OR neo-natal* OR postnatal* OR post-natal* ~~OR pregnan* OR fetus* OR fetus* OR fetal* OR fetal*~~ OR baby OR babies OR neonate* OR neo-nate* OR newborn* OR new-born* OR infant*).ti,ab. OR infant/ OR infant, newborn/ OR infant, low birth weight/ OR infant, small for gestational age/ OR infant, very low birth weight/ OR infant, extremely low birth weight/ OR infant, postmature/ OR infant, premature/ OR infant, extremely premature/ OR birth weight/

No other filters or limits were applied. For the Embase search, all MeSH terms were manually translated to their corresponding Emtree terms. For the Google Scholar, ClinicalTrials.gov, and ICTRP searches, the original MEDLINE search was manually shortened to fit the specific character limits and other platform restrictions.

Here, we present all eight search strategies in full:

1. MEDLINE
2. Embase
3. CINAHL
4. Web Of Science Core Collection
5. Scopus
6. Google Scholar
7. ClinicalTrials.gov
8. WHO ICTRP

1. MEDLINE (Ovid Technologies, Inc)

1 infant/ or infant, newborn/ or infant, low birth weight/ or infant, small for gestational age/ or infant, very low birth weight/ or infant, extremely low birth weight/ or infant, postmature/ or infant, premature/ or infant, extremely premature/ or birth weight/ or (perinatal* or neonatal* or neo-natal* or postnatal* or post-natal* or baby or babies or neonate* or neo-nate* or newborn* or new-born* or infant*).ti,ab.

2 pain/ or acute pain/ or nociceptive pain/ or pain, postoperative/ or pain, procedural/ or pain perception/ or nociception/ or hyperalgesia/ or pain measurement/ or pain management/ or pain threshold/ or nociceptors/ or analgesia/ or (pain* or nocicept* or noxious* or allodynia* or allo-dynia* or hyperalges* or hyper-alges* or hypoalges* or hypo-alges* or analges*).ti,ab.

3 electroencephalography/ or evoked potentials/ or laser-evoked potentials/ or cortical synchronization/ or electroencephalography phase synchronization/ or brain waves/ or alpha rhythm/ or beta rhythm/ or delta rhythm/ or gamma rhythm/ or theta rhythm/ or cortical excitability/ or (eeg* or electroencephalogra* or electro-encephalogra* or erp or event-related potential* or event related potential* or evoked response* or brain activit* or brain function* or cortical activit* or cortical function*).ti,ab.

4 1 and 2 and 3

2. Embase (Ovid Technologies, Inc)

1 infant/ or baby/ or high risk infant/ or hospitalized infant/ or newborn/ or low birth weight/ or small for date infant/ or very low birth weight/ or extremely low birth weight/ or postmaturity/ or prematurity/ or birth weight/ or high birth weight/ or (perinatal* or neonatal* or neo-natal* or postnatal* or post-natal* or baby or babies or neonate* or neo-nate* or newborn* or new-born* or infant*).ti,ab.

2 pain/ or allodynia/ or mechanical allodynia/ or tactile allodynia/ or thermal allodynia/ or cold allodynia/ or heat allodynia/ or experimental pain/ or hyperalgesia/ or mechanical hyperalgesia/ or opioid induced hyperalgesia/ or thermal hyperalgesia/ or cold hyperalgesia/ or heat hyperalgesia/ or hypoalgesia/ or thermal hypoalgesia/ or inflammatory pain/ or injection pain/ or injection site pain/ or nociceptive pain/ or postoperative pain/ or procedural pain/ or pain assessment/ or behavioral pain scale/ or nociception/ or gate control theory/ or nociceptive stimulation/ or pain threshold/ or heat pain threshold/ or pressure pain threshold/ or pain receptor/ or pain measurement/ or algometry/ or analgesia/ or antinociception/ or epidural analgesia/ or postoperative analgesia/ or (pain* or nocicept* or noxious* or allodynia* or allo-dynia* or hyperalges* or hyper-alges* or hypoalges* or hypo-alges* or analges*).ti,ab.

3 electroencephalography/ or continuous electroencephalography/ or electroencephalography monitoring/ or electroencephalogram/ or event related potential/ or evoked response/ or evoked cortical response/ or alpha rhythm/ or beta rhythm/ or delta rhythm/ or gamma rhythm/ or theta rhythm/ or hippocampus theta rhythm/ or mu rhythm/ or cortical excitability/ or laser evoked potential/ or cortical synchronization/ or electroencephalography phase synchronization/ or (eeg* or electroencephalogra* or electro-encephalogra* or erp or event-related potential* or event related potential* or

evoked response* or brain activit* or brain function* or cortical activit* or cortical function*).ti,ab.

4 1 and 2 and 3

3. CINAHL (EBSCO Industries)

S1 (MH infant) OR (MH "infant, newborn") OR (MH "infant, low birth weight") OR (MH "infant, small for gestational age") OR (MH "infant, very low birth weight") OR (MH "infant, extremely low birth weight") OR (MH "infant, postmature") OR (MH "infant, premature") OR (MH "infant, extremely premature") OR (MH "birth weight") OR ((TI perinatal* OR AB perinatal*) OR (TI neonatal* OR AB neonatal*) OR (TI neo-natal* OR AB neo-natal*) OR (TI postnatal* OR AB postnatal*) OR (TI post-natal* OR AB post-natal*) OR (TI baby OR AB baby) OR (TI babies OR AB babies) OR (TI neonate* OR AB neonate*) OR (TI neo-nate* OR AB neo-nate*) OR (TI newborn* OR AB newborn*) OR (TI new-born* OR AB new-born*) OR (TI infant* OR AB infant*))

S2 (MH pain) OR (MH "acute pain") OR (MH "nociceptive pain") OR (MH "pain, postoperative") OR (MH "pain, procedural") OR (MH "pain perception") OR (MH nociception) OR (MH hyperalgesia) OR (MH "pain measurement") OR (MH "pain management") OR (MH "pain threshold") OR (MH nociceptors) OR (MH analgesia) OR ((TI pain* OR AB pain*) OR (TI nocicept* OR AB nocicept*) OR (TI noxious* OR AB noxious*) OR (TI allodynia* OR AB allodynia*) OR (TI allo-dynia* OR AB allo-dynia*) OR (TI hyperalges* OR AB hyperalges*) OR (TI hyper-alges* OR AB hyper-alges*) OR (TI hypoalges* OR AB hypoalges*) OR (TI hypo-alges* OR AB hypo-alges*) OR (TI analges* OR AB analges*))

S3 (MH electroencephalography) OR (MH "evoked potentials") OR (MH "laser-evoked potentials") OR (MH "cortical synchronization") OR (MH "electroencephalography phase synchronization") OR (MH "brain waves") OR (MH "alpha rhythm") OR (MH "beta rhythm") OR (MH "delta rhythm") OR (MH "gamma rhythm") OR (MH "theta rhythm") OR (MH "cortical excitability") OR ((TI eeg* OR AB eeg*) OR (TI electroencephalogra* OR AB electroencephalogra*) OR (TI electro-encephalogra* OR AB electro-encephalogra*) OR (TI erp OR AB erp) OR (TI "event-related potential*" OR AB "event-related potential*") OR (TI "event related potential*" OR AB "event related potential*") OR (TI "evoked response*" OR AB "evoked response*") OR (TI "brain activit*" OR AB "brain activit*") OR (TI "brain function*" OR AB "brain function*") OR (TI "cortical activit*" OR AB "cortical activit*") OR (TI "cortical function*" OR AB "cortical function*"))

S4 S1 AND S2 AND S3

4. Web Of Science Core Collection (Clarivate Analytics)

1 ALL=infant OR ALL="infant, newborn" OR ALL="infant, low birth weight" OR ALL="infant, small for gestational age" OR ALL="infant, very low birth weight" OR ALL="infant, extremely low birth weight" OR ALL="infant, postmature" OR ALL="infant, premature" OR ALL="infant, extremely premature" OR ALL="birth weight" OR (TI=(perinatal* OR neonatal* OR neo-natal* OR postnatal* OR post-natal* OR baby OR babies OR neonate* OR neo-nate* OR newborn* OR new-born* OR infant*) OR AB=(perinatal* OR neonatal* OR neo-natal* OR postnatal* OR post-natal* OR baby OR babies OR neonate* OR neo-nate* OR newborn* OR new-born* OR infant*))

2 ALL=pain OR ALL="acute pain" OR ALL="nociceptive pain" OR ALL="pain, postoperative" OR ALL="pain, procedural" OR ALL="pain perception" OR ALL=nociception OR ALL=hyperalgesia OR ALL="pain measurement" OR ALL="pain management" OR ALL="pain threshold" OR ALL=nociceptors OR ALL=analgesia OR (TI=(pain* OR nocicept* OR noxious* OR allodynia* OR allo-dynia* OR hyperalges* OR hyper-alges* OR hypoalges* OR hypo-alges* OR analges*) OR AB=(pain* OR nocicept* OR noxious* OR allodynia* OR allo-dynia* OR hyperalges* OR hyper-alges* OR hypoalges* OR hypo-alges* OR analges*))

3 ALL=electroencephalography OR ALL="evoked potentials" OR ALL="laser-evoked potentials" OR ALL="cortical synchronization" OR ALL="electroencephalography phase synchronization" OR ALL="brain waves" OR ALL="alpha rhythm" OR ALL="beta rhythm" OR ALL="delta rhythm" OR ALL="gamma rhythm" OR ALL="theta rhythm" OR ALL="cortical excitability" OR (TI=(eeg* OR electroencephalogra* OR electro-encephalogra* OR erp OR "event-related potential*" OR "event related potential*" OR "evoked response*" OR "brain activit*" OR "brain function*" OR "cortical activit*" OR "cortical function*") OR AB=(eeg* OR electroencephalogra* OR electro-encephalogra* OR erp OR "event-related potential*" OR "event related potential*" OR "evoked response*" OR "brain activit*" OR "brain function*" OR "cortical activit*" OR "cortical function*"))

4 #1 AND #2 AND #3

5. Scopus (Elsevier)

(INDEXTERMS(infant) OR INDEXTERMS("infant, newborn") OR INDEXTERMS("infant, low birth weight") OR INDEXTERMS("infant, small for gestational age") OR INDEXTERMS("infant, very low birth weight") OR INDEXTERMS("infant, extremely low birth weight") OR INDEXTERMS("infant, postmature") OR INDEXTERMS("infant, premature") OR INDEXTERMS("infant, extremely premature") OR INDEXTERMS("birth weight") OR TITLE-ABS(perinatal* OR neonatal* OR neo-natal* OR postnatal* OR post-natal* OR baby OR babies OR neonate* OR neo-nate* OR newborn* OR new-born* OR infant*)) AND (INDEXTERMS(pain) OR INDEXTERMS("acute pain") OR INDEXTERMS("nociceptive pain") OR INDEXTERMS("pain, postoperative") OR INDEXTERMS("pain,

procedural") OR INDEXTERMS("pain perception") OR INDEXTERMS(nociception) OR INDEXTERMS(hyperalgesia) OR INDEXTERMS("pain measurement") OR INDEXTERMS("pain management") OR INDEXTERMS("pain threshold") OR INDEXTERMS(nociceptors) OR INDEXTERMS(analgesia) OR TITLE-ABS(pain* OR nocicept* OR noxious* OR allodynia* OR allo-dynia* OR hyperalges* OR hyper-alges* OR hypoalges* OR hypo-alges* OR analges*)) AND (INDEXTERMS(electroencephalography) OR INDEXTERMS("evoked potentials") OR INDEXTERMS("laser-evoked potentials") OR INDEXTERMS("cortical synchronization") OR INDEXTERMS("electroencephalography phase synchronization") OR INDEXTERMS("brain waves") OR INDEXTERMS("alpha rhythm") OR INDEXTERMS("beta rhythm") OR INDEXTERMS("delta rhythm") OR INDEXTERMS("gamma rhythm") OR INDEXTERMS("theta rhythm") OR INDEXTERMS("cortical excitability") OR TITLE-ABS(eeg* OR electroencephalogra* OR electro-encephalogra* OR erp OR "event-related potential*" OR "event related potential*" OR "evoked response*" OR "brain activit*" OR "brain function*" OR "cortical activit*" OR "cortical function*"))

6. Google Scholar (Publish or Perish)

Keywords:
(perinatal OR neonatal OR postnatal OR baby OR babies OR neonate OR newborn OR infant) AND (pain OR nociception OR noxious OR analgesia OR analgesic) AND (eeg OR electroencephalography OR electroencephalographic OR erp OR "event related potential")

Maximum number of results:
200

7. ClinicalTrials.gov (https://clinicaltrials.gov)

Search 1: pain AND babies
Status:
“All studies”

“Condition or disease”:
pain OR nociception OR noxious OR allodynia OR hyperalgesia OR hypoalgesia OR analgesia OR allodynic OR hyperalgesic OR hypoalgesic OR analgesic

“Other terms”:
perinatal OR neonatal OR postnatal OR baby OR babies OR neonate OR newborn OR infant

Search 2: pain AND EEG
Status:
“All studies”

“Condition or disease”:
pain OR nociception OR noxious OR allodynia OR hyperalgesia OR hypoalgesia OR analgesia OR allodynic OR hyperalgesic OR hypoalgesic OR analgesic

“Other terms”:
eeg OR electroencephalography OR electroencephalographic OR erp OR "event-related potential" OR "event related potential" OR "evoked response" OR "brain activity" OR "brain function" OR "cortical activity" OR "cortical function"

Intersection of searches 1 and 2: pain AND babies AND EEG
Performed external to registry using python (code for this step is publicly available here: https://github.com/lukebax/clinical-trial-registry-data-wrangling

8. WHO ICTRP (https://trialsearch.who.int)

Search 1: pain
pain OR nociception OR noxious OR analgesia OR analgesic

Search 2: babies
infant OR newborn OR neonate OR baby

Intersection of searches 1 and 2: pain AND babies
Performed external to registry using python (code for this step is publicly available here: https://github.com/lukebax/clinical-trial-registry-data-wrangling

Risk of Bias in Individual Studies

Novel outcomes and by-site grouping

The purpose of this review is to assess basic properties of an analysis method that will not have been used in several of the relevant available publications. The existing publications that are relevant to this review were identified by their use of relevant data, not by their reporting of relevant outcomes. As such, the outcomes of interest for this review cannot be derived from or compared to those of the relevant available publications. We will have to return to the original raw EEG data to generate the relevant outcomes, which will be a set of novel outcomes that assess our analysis methodology. Given that traditional risk of bias assessments focus on existing reports (published or grey literature) and assess bias at the outcome-level [10] or the study-level [11], these methods are not directly applicable to this review. We will thus not apply standardised checklists for risk of bias assessments. Instead, we list here the set of topics relevant to bias and quality assessments and outline our strategy to assess them.

Due to the small sample sizes that are typical of EEG studies of neonatal pain, the common practice of reusing a subject's data in distinct publications to derive maximum value from limited but precious neonatal EEG databases, and the public availability of a relevant neonatal EEG database from the University College London site [12], we will group our two-stage individual participant data (IPD) meta-analyses by site (or adjust our one-stage IPD meta-analyses for site). Grouping by site will help to avoid duplications of subject data that may occur across publications; it will ensure reasonable sample sizes per site dataset; it will allow cross-site comparisons of primary outcomes (i.e. validity, reliability, and interpretability); and it should facilitate site dataset standardisation and sharing by participating original investigators.

Standardised checklists and RCT randomisation integrity:

Due to the novel outcomes and by-site grouping of this review, assessing the randomisation integrity of clinical trials and applying standardised risk of bias checklists to included studies or published outcomes is not applicable to this review.

Availability bias:

Availability bias is bias due to differences between which data were collected and which were provided in the IPD (e.g. if outcomes with negative results were not provided by the original investigators) [13]. Availability bias due to missing data will be assessed by comparing provided IPD with the data descriptions in the relevant available study records (e.g. protocols, trial registry reports, published articles) and discussion with original investigators.

Data accuracy:

To assess data accuracy, we will identify, assess, and resolve (if possible) (i) duplicates and (ii) nonsensical values that lie outside reasonable ranges.

Data quality assessment:

As part of EEG data quality assessment, we will quantify the proportion of EEG epochs rejected due to data quality issues (e.g. electrode pop-off).

Small study effects, identified using graphical and statistical methods, are often used to assess publication bias (see discussion of publication bias below). Given our grouping of data by site, we will explore "small site effects" using graphical methods (such as funnel plots) and statistical methods (such as Egger's test), if datasets from 10 sites or more are available in our review. While this is a non-standard use of such methods, exploring the relationship between a site's sample size and the site's effect size may shed light on data quality and risk of bias. However, this assessment will be exploratory and strong conclusions should not be expected. This assessment can be performed for primary outcomes (measures of validity, reliability, and interpretability) as well as measures of data quality (e.g. proportion of trials rejected).

Meta-bias(es)

Risk of bias versus meta-bias:
Procedural problems during the conduct of a primary study, such as inappropriate method of random sequence generation in randomized trials, result in methodological biases. Meta-biases occur independent of procedural problems during the conduct of a primary study, and the two meta-biases outlined in PRISMA-P [14, 15] are outcome reporting bias and publication bias. Due to the novel outcomes and by-site grouping of this review, traditional methods for assessing meta-bias are not directly applicable to this review.

Outcome reporting bias:
Outcome reporting bias occurs when there is selective reporting of outcomes. In aggregate data meta-analyses, outcome reporting bias can be identified by spotting mismatches between what outcomes get reported in the results section of a publication and what outcomes were described in the methods section of the publication or the associated protocol (if available). Our review outcomes will differ from those of the original investigators and those published as outcomes in the relevant literature. Thus, assessing outcome reporting bias is not applicable to this review. A related bias called availability bias is relevant to our review, and details regarding our assessment of availability bias are provided above.

Publication bias:
Publication bias occurs when the likelihood of a study being published is affected by the findings of the study. Our primary strategy for addressing publication bias is to avoid limiting data requests to data identified only in published records: we will be requesting data described in records published in both peer-reviewed journals as well as grey literature. Publication bias is known to result in small study effects (although evidence of small study effects is not conclusive evidence of publication bias). Given our outcomes of interest are novel and will not be published outcomes, and given our grouping by site rather than by publication, we will not be assessing small study effects as it is not applicable to this review. A related effect that we call "small site effects"

(exploring the relationship between a site's sample size and the site's effect size) may be relevant to identifying issues with data quality or bias, and details regarding our exploration of small site effects are provided above.

Supplementary References

1. EMA. Investigation of medicinal products in the term and preterm neonate. European Medicines Agency. 2009. https://www.ema.europa.eu/en/investigation-medicinal-products-term-preterm-neonate. Accessed 1 Aug 2022.

2. EMA. ICH E11(R1) step 5 guideline on clinical investigation of medicinal products in the pediatric population. European Medicines Agency. 2017. https://www.ema.europa.eu/en/ich-e11r1-step-5-guideline-clinical-investigation-medicinal-products-pediatric-population. Accessed 1 Aug 2022.

3. IASP. IASP Terminology - IASP. 2022. https://www.iasp-pain.org/resources/terminology/. Accessed 13 Jun 2020.

4. Walco GA, Kopecky EA, Weisman SJ, Stinson J, Stevens B, Desjardins PJ, et al. Clinical trial designs and models for analgesic medications for acute pain in neonates, infants, toddlers, children, and adolescents: ACTTION recommendations. Pain. 2018;159:193–205.

5. Walton MK, Powers JH, Hobart J, Patrick D, Marquis P, Vamvakas S, et al. Clinical Outcome Assessments: Conceptual Foundation—Report of the ISPOR Clinical Outcomes Assessment – Emerging Good Practices for Outcomes Research Task Force. Value in Health. 2015;18:741–52.

6. FDA-NIH Biomarker Working Group. BEST (Biomarkers, EndpointS, and other Tools) Resource. Silver Spring (MD): Food and Drug Administration (US); 2016.

7. De Vet HCW, Terwee CB, Mokkink LB, Knol DL. Measurement in Medicine: A Practical Guide. Cambridge: Cambridge University Press; 2011.

8. Mokkink LB, Terwee CB, Patrick DL, Alonso J, Stratford PW, Knol DL, et al. The COSMIN study reached international consensus on taxonomy, terminology, and definitions of measurement properties for health-related patient-reported outcomes. J Clin Epidemiol. 2010;63:737–45.

9. Davidson M. Known-Groups Validity. In: Michalos AC, editor. Encyclopedia of Quality of Life and Well-Being Research. Dordrecht: Springer Netherlands; 2014. p. 3481–2.

10. Higgins J, Savović J, Page M, Elbers R, Sterne J. Chapter 8: Assessing risk of bias in a randomized trial. In: Higgins J, Thomas J, Chandler J, Cumpston M, Li T, Page M, et al., editors. Cochrane Handbook for Systematic Reviews of Interventions. version 6.4 (updated August 2023). Cochrane; 2023.

11. Barker TH, Stone JC, Sears K, Klugar M, Leonardi-Bee J, Tufanaru C, et al. Revising the JBI quantitative critical appraisal tools to improve their applicability: an overview of methods and the development process. JBI Evid Synth. 2022. https://doi.org/10.11124/JBIES-22-00125.

12. Jones L, Laudiano-Dray MP, Whitehead K, Verriotis M, Meek J, Fitzgerald M, et al. EEG, behavioural and physiological recordings following a painful procedure in human neonates. Sci Data. 2018;5:180248.

13. Banerjee A, Nunan D. Availability bias. In: Catalogue of Bias Collaboration, editor. Catalogue of Bias. 2019.

14. Moher D, Shamseer L, Clarke M, Ghersi D, Liberati A, Petticrew M, et al. Preferred reporting items for systematic review and meta-analysis protocols (PRISMA-P) 2015 statement. Systematic Reviews. 2015;4:1.

15. Shamseer L, Moher D, Clarke M, Ghersi D, Liberati A, Petticrew M, et al. Preferred reporting items for systematic review and meta-analysis protocols (PRISMA-P) 2015: elaboration and explanation. BMJ. 2015;349:g7647.
